# Enteroviruses and Adenoviruses in stool specimens of paralytic children- can they be the cause of paralysis?

**Published:** 2018-06

**Authors:** Maryam Yousefi, Ahmad Nejati, Seyed Mohsen Zahraei, Sussan Mahmoudi, Najmeh Parhizgari, Seyed Mohammad Jazayeri Farsani, Mahmood Mahmoodi, Rakhshandeh Nategh, Shohreh Shahmahmoodi

**Affiliations:** 1 Virology Department, School of Public Health, International Campus, Tehran University of Medical Sciences, Tehran, Iran; 2 Virology Department, School of Public Health, Tehran University of Medical Sciences, Tehran, Iran; 3 Vaccine Preventable Diseases Department, Center or Communicable Diseases Control, Ministry of Health and Medical Education, Tehran, Iran; 4 Laboratory of Experimental Virology, Department of Medical Microbiology, Center for Infection and Immunity Amsterdam Academic Medical Center, University of Amsterdam, Netherlands; 5 Department of Epidemiology and Biostatistics, School of Public Health, Tehran University of Medical Sciences, Tehran, Iran; 6 Food Microbiology Research Center, Tehran University of Medical Sciences, Tehran, Iran

**Keywords:** Acute flaccid paralysis, Residual paralysis, Non-polio *Enterovirus*, *Adenovirus*

## Abstract

**Background and Objectives:**

Acute flaccid paralysis (AFP) is a complicated clinical syndrome with a wide range of potential etiologies. Several infectious agents including different virus families have been isolated from AFP cases. In most surveys, Non-polio Enteroviruses (NPEVs) have been detected as main infectious agents in AFP cases; however, there are also some reports about *Adenovirus* isolation in these patients. In this study, NPEVs and *Adenoviruses* in stool specimens of AFP cases with or without Residual Paralysis (RP) with negative results for poliovirus are investigated.

**Materials and Methods:**

Nucleic acid extractions from 55 AFP cases were examined by nested PCR or semi-nested PCR with specific primers to identify NPEVs or *Adenoviruses*, respectively. VP1 (for *Enteroviruses*) and hexon (for *Adenoviruses*) gene amplification products were sequenced and compared with available sequences in the GenBank.

**Results:**

From 55 fecal (37 RP+ and 18 RP−) specimens, 7 NPEVs (12.7%) (2 cases in RP+) and 7 *Adenoviruses* (12.7%) (4 cases in RP+) were identified. *Echovirus* types 3, 17 and 30, *Coxsackie virus* A8, and *Enterovirus* 80 were among NPEVs and *Adenoviruses* type 2 and 41 were also identified.

**Conclusion:**

Our finding shows that NPEVs and *Adenoviruses* may be isolated from the acute flaccid paralyses but there is no association between the residual paralyses and virus detection.

## INTRODUCTION

Acute flaccid paralysis (AFP) is known as a sudden onset of flaccid paralysis in one or more limbs in children less than 15 years old (1). Infectious and non-infectious agents, (e.g. metabolic disorders), trauma and metal toxicity as well as post-infectious autoimmune conditions (e.g. Guillain-Barre Syndrome) may be involved in the establishment of this syndrome in humans. A group of AFP patients suffer from Residual Paralysis (RP) and the rest recover. Several studies have been performed on patients who are suffering from RP (2, 3). Among infectious agents, viruses from different families such as *Enteroviruses, Flaviviruses, Herpes viruses, Rabies virus* and some *Arboviruses* have been isolated frequently from stool samples of AFP (4, 5). Different cases of AFP caused by various agents cannot be distinguished only by their clinical symptoms (5).

Currently, as a part of the Global Polio Laboratory Network (GPLN) program, AFP is monitored in countries where poliovirus is still endemic (6). Wild-type and vaccine-derived strains of polio and many NPEVs are frequently isolated in AFP cases (7, 8). Based on WHO recommendations, Iran conducts AFP surveillance in children under 15 years of age to screen for the poliovirus. The surveillance includes follow-up of the AFP cases 60 days after paralysis onset to detect RP.

*Enteroviruses* are ubiquitous viruses that infect all populations worldwide. About 100 distinct serotypes of Human *Enteroviruses* (HEV) have been classified into four species including Poliovirus (PV), HEV-A, B, C and D (9). The genome contains about 7,500 nucleotides, and it encodes the same set of proteins with strong sequence conservation. *Enteroviruses* are responsible for a variety of clinical syndromes such as paralytic poliomyelitis, myocarditis, acute hemorrhagic conjunctivitis, undifferentiated rash, and the common cold (10).

*Adenoviruses* are non-enveloped dsDNA viruses. To date, about 85 genotypes of Human *Adenoviruses* (HAdV) are classified into seven species of HAdV-A to HAdV-G in the genus *Mastadenovirus* (11). *Adenovirus* infections are usually asymptomatic although different HAdV-B, HAdV-C, and HAdV-E types cause mild respiratory syndromes (12). HAdV species are also involved in the development of conjunctivitis, pneumonia, gastroenteritis, hemorrhagic cystitis, myocarditis, and systemic infection (13). *Adenoviruses* have also been shown to infect the Central Nervous System (CNS). They have been detected in the spinal cord and Cerebrospinal Fluid (CSF) of patients with neurological manifestations (14, 15) and have been isolated from patients with acute myelopathies (16) and AFP (17).

This study aimed to detect NPEVs as well as *Adenoviruses* in AFP cases with or without RP to find any probable correlation between viral infection and flaccid paralysis.

## MATERIALS AND METHODS

### Specimens and sample preparation.

We used fifty-five patients with clinical features of AFP referred to the Iran National Polio Laboratory (INPL), all less than 15 years of age and recipients of at least 3 doses of oral polio vaccine (OPV). All cases were tracked for 60 days in order to investigate RP status. Stool specimens were subjected to chloroform pre-treatment. Briefly, about 2 gr of each sample was mixed with 9 ml of Phosphate Buffer Saline (PBS) and 1 ml of chloroform. The solution was shaken vigorously for 20 minutes and centrifuged (20 min, 2100 ×g, 4°C) (5). The supernatant was used for nucleic acid extraction.

### RNA and DNA extraction.

High pure viral nucleic acid kit (Roche Applied Science; Mannheim, Germany) was used to extract viral DNA and RNA according to manufacturer’s instructions. The concentration and purity of the extracted nucleic acid were determined by using the NanoDrop ND-1000 Spectrophotometer (NanoDrop Technologies, Wilmington, DE, USA).

### Nested RT-PCR for NPEVs.

cDNA synthesis was performed using Reverse Transcriptase AMV kit (Roche Applied Science; Mannheim, Germany) based on the manufacturer’s instructions. Nested PCR was performed with a first-round PCR by 224/222 primers (5′-GCIATGYTIGGIACICAYRT-3′/5′-CICCIGGIGGIAYRWACAT-3′) for amplification of 762 bps at the intersection of the 3′ end of VP3 and 5′ end of VP1 region. Subsequently, a second-round of PCR was performed using AN89/AN88 primers (5′-CCAGCACTGACAGCAGYNGARAYNGG-3′/5′-TACTGGACCACCTGGNGGNAYRWACAT-3′) for amplification of a 372 bp region in the VP1 gene. The reaction was performed in a final volume of 25 μl. Each mixture contained 2.5 μL of 10X PCR buffer, 0.2 mM deoxynucleotide triphosphates (dNTPs), 1 μl of Hot start Taq DNA polymerase (Qiagen, California, USA), 0.5 μM of primers 224/222 and double distilled water. Five microliters of the cDNA serving as the template for the first-round of PCR. The second-round of PCR was performed using 1 μl of the first-round PCR product as template and primers AN89/AN88 for amplification of 372 bp product in VP1 gene.

The following cycling profile was used for amplification: a 3-minute initial denaturation step at 94°C, 35 cycles of 30 seconds for denaturation at 94°C, 30 seconds annealing at 44°C (for the first-round) or 51°C (for the second-round), and 30 seconds extension at 72°C. The last cycle was followed by a final extension step of 5 minutes at 72°C.

### Semi-nested PCR for Adenoviruses.

Semi-nested PCR was performed with a first-round PCR reaction using Adf/AdR1 primers for amplification of 243 bp in the hexon gene followed by a second-round PCR using Adf/AdR2a and AdR2b primers for amplification of 227 bp region in hexon gene. The reaction was performed in a final volume of 50 μl. Each mixture contained 5 μl of 10X PCR buffer, 0.2 μM deoxy-nucleotide triphosphates (dNTPs), 1.5 mM MgCl_2_, 1 U Hotstart Taq DNA polymerase (Qiagen, California, USA), 0.5 μM of Adf/AdR1 primers and double distilled water. Ten microliters of the extracted DNA and 5 μl of the first-round PCR product served as templates for the first-round and second-round PCRs, respectively.

The following cycling profile was used for amplification: a Five-minute initial denaturation step at 94°C, 30 cycles of 30 seconds denaturation at 94°C, 25 seconds annealing at 56°C, and 15 seconds extension at 72°C. The last cycle was followed by a final extension step of 8 minutes at 72°C.

### DNA sequencing and phylogeny.

All of the amplified PCR products were analyzed by electrophoresis in 2% agarose gel stained with SimplySafe (EURx, Poland) and visualized under UV light. PCR products were then extracted and purified from the gel using QIAquick gel extraction kit (Qiagen Ltd, Crawley, UK). The second round PCR products of *Enteroviruses* and *Adenoviruses* were sequenced using the ABI 3110 DNA SEQUENCER (Applied Biosystems, Foster City, CA). The partial nucleotide sequences of VP1 and hexon regions were aligned by the CLUSTALW and phylogenetic tree analysis was obtained by the neighbor-joining method under the Kimura-two-parameter distance model by the software package of MEGA7 (www.megasoftware.net). The reference strains used for comparisons with sequences from this study were obtained from GenBank.

In order to identify *Enterovirus* serotypes, pairwise comparison of the VP1 amplicon sequence was performed with the complete database of VP1 sequences of all known EV serotypes using the BLAST program (www.ncbi.nlm.gov/BLAST) from the Gen-Bank. At least 75% identity in the VP1 sequence is required to put strains in the same serotype (18).

Types of *Adenoviruses* were determined by comparing the sequences obtained from the hexon amplified region with the sequences of the same region of prototype strains existing in the GenBank database

## RESULTS

Out of all referred stool specimens from AFP cases referred to the Iran National Polio Laboratory (INPL) during 2015–2016, fifty-five were randomly selected to search for *Enterovirus* and *Adenovirus* infection. The age of examined cases ranged from 1 to 14.5 years with a mean of 5.5 years. A total of 35 out of 55 subjects (63.6%) were male. The highest virus isolation rate was observed among children aged less than 5 years old.

After 60 days of following the paralysis status, 37 out of 55 AFP cases were confirmed to have RP. Virological investigation among RP+ has confirmed the existence of one *Adenovirus* type 2, two *Adenovirus* type 41, one *Coxsackievirus* A8 and a coinfection of *Adenovirus* 2 and *Enterovirus* B80. In addition, the results showed three *Adenovirus* 41, two *Echovirus* 17, an *Echovirus* 30, an *Echovirus* 3 and two *Coxsackievirus* A8 in Non-Residual Paralysis patients (Table 1).

**Table 1.** Demographic information from patients with *Enterovirus* or *Adenovirus* infection

	**RP status**	**Sex**	**Age (year)**	**PCR detection**	**Sequence (Identity)**	**Result**
1	+	Male	5	*Entrovirus*	(%82)	*Coxackivirus* A8
2	−	Male	3	*Entrovirus*	(%90)	*Coxackivirus* A8
3	−	Male	3	*Entrovirus*	(%92)	*Echovirus* 17
4	−	Male	3	*Entrovirus*	(%90)	*Echovirus* 17
5	−	Female	2	*Entrovirus*	(%89)	*Echovirus* 3
6	−	Female	2	*Entrovirus*	(%89)	*Echovirus* 30
7	+	Male	1	*Adenovirus*	(%90)	*Adenovirus* 41
8	+	Male	4.5	*Adenovirus*	(%98)	*Adenovirus* 41
9	+	Male	2	*Adenovirus*	(%92)	*Adenovirus* 2
10	−	Male	5	*Adenovirus*	(%98)	*Adenovirus* 41
11	−	Female	1.5	*Adenovirus*	(%98)	*Adenovirus* 41
12	−	Female	1	*Adenovirus*	(%99)	*Adenovirus* 41
13	+	Male	1	*Adenovirus*	(%99)*	*Adenovirus* 2
				*Entrovirus*	(%92)*	*Enterovirus* B80

* A case of co-infection

Altogether, among 55 fecal samples of AFP patients, 7 NPEVs (12.72%) and 7 *Adenoviruses* (12.72%) were identified by PCR. The 7 *Enteroviruses* detected were as follow: *Echo-17, Echo-30, Echo-E3,* two *Coxsackievirus* type A8, and one *Enterovirus* B80. Moreover, from a total of 7 *Adenoviruses* determined by PCR, *Adenovirus* 41 and *Adenovirus* 2 were found in 5 and 2 patients, respectively.

**Fig. 1.**
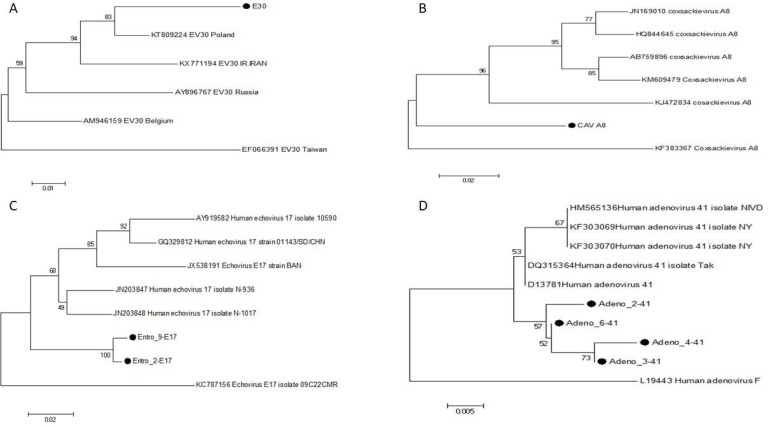
Phylogenetic trees showing genetic relationships Figure Legend: Phylogenetic tree of *Enteroviruses* and *Adenoviruses* from Iran and reference sequences from GenBank. The tree was constructed by the neighbor-joining algorithm implemented in the MEGA-7 program using p-Distance. Sequences from Iran are indicated by black circles, and reference sequences are indicated by accession number. (A) Human *Echovirus* 30, (B) Human *Coxsackievirus* A8, (C) Human *Echovirus* 17, (D) Human *Adenovirus* 41.

To consider the genetic similarity of *Enterovirus* and *Adenovirus* isolates in this study with worldwide circulating types, phylogenetic analysis of partial VP1 of *Echovirus* 17, human *Coxsackievirus* A8 and *Echovirus* 30 and hexon of *Adenovirus* 41 were performed. The results of *Echovirus* 17, human *Coxsackievirus* A8 and *Adenovirus* 41 showed there is a different cluster in comparison to other strains. However, *Echovirus* 30 showed a similarity between Poland strain and previous Iran strain (19) (Fig. 1).

## DISCUSSION

The poliomyelitis eradication program has progressed by AFP surveillance. The main aim of AFP surveillance is the detection of poliovirus, but neurological diseases can be induced by non-infectious or infectious pathogens other than poliovirus. Some known viral families have been repeatedly detected in stool specimens of AFP cases. However, their etiologic role need to be further studied. In this study, the presence of *Enterovirus* and *Adenovirus* nucleic acid was investigated in stool specimens of 55 RP+ and RP- children with confirmed Non-polio AFP. Sequencing results confirmed the presence of NPEVs and *Adenoviruses* in 13 out of 55 cases. NPEV infection was 12.72% in studied specimens, which is comparable with the results of Bingjun et al. which reported 17.2% NPEV infection among AFP cases (20). However, an investigation in healthy children showed that infection with NPEVs circulates commonly in all populations (21, 22), so NPEV detection cannot be always attributed to the symptoms of AFP. On the contrary, an association has been found between NPEV detection (e.g., *Coxsackievirus* A7 and *Enterovirus* 71) and both paralytic disease and outbreaks that have clinically mimicked poliomyelitis (23, 24).

This is the first report of detection of *Echovirus* type 17, and *Coxsackievirus* A8 and *Adenovirus* type 2 in stool specimens of children with AFP in Iran. Interestingly, a co-infection of *Enterovirus* 80 and *Adenovirus* type 2 was also found in a one year old boy, although, detection of *Enterovirus* 80 has been reported in China, Nigeria, Philippines, Oman, Kenya, and India (25, 26, 27).

Rahimi et al. reported the detection of *Enteroviruses* in patients with RP during 2002–2003 in 29 stool specimens in Iran. They found different serotypes of *Echoviruses* such as *Echovirus* serotypes 3, 4, 5, 6, 7, 9, 12, 18 and 33. This study showed the presence of *Adenovirus* 41, *Adenovirus* 2, *Enterovirus* B80 and *Coxsackievirus* A8 nucleic acid in children suffering from RP. The results indicated that new serotypes have been isolated in comparison with previous studies (28). Moreover, the isolation rate of *Adenoviruses* in the current study was 12.7% which is higher than the isolation rates of 1.9% and 1.05% reported in Brazil and Russia, respectively. There are some reports which have mentioned that HAdV-B species have neuropathogenic potential and they could be responsible for a fraction of AFP cases. In this study, HAdV-C2 and HAdV-F41 types were only found with no significant statistical correlation between *Adenovirus* infection and RP. Although HAdV-C2 has been isolated from both nasopharyngeal and fecal specimens, *Adeno-F41* has only been detected in stool specimens of children with gastroenteritis (29). *Adenovirus* infections are ubiquitous and their clinical manifestations are usually subclinical or very mild. Detection of *Adenoviruses* cannot prove the etiological role of these viruses in AFP even if they really induce neuropathogenic effects. On the other hand, isolation of the same virus from many healthy patients does not rule out its capacity to cause the disease. However, detection of the virus in cerebrospinal fluid can be more supportive of its role in AFP. Dhole et al. in India have reported *Cox*-B, *Echo*-11, *Echo*-12, *Echo*-13, *Echo*-7, *Echo*-20, *Echo*-14, *Echo-*30 as the main serotypes detected in AFP stool specimens during 2004–2007 while results of this study showed a different pattern of viral infection in this group (10). Observation of totally different serotypes appears to be logical as there is a big difference between environmental health circumstances, geographical location, viral epidemics and viruses which are endemic in the countries. These circumstances could influence viral infections in different countries. Phylogenetic analysis of *Echovirus* 17, human *Coxsackievirus* A8 and *Adenovirus* 41 indicated these viruses have been genetically distinct from other strains. Thus, this study highlighted different virus strains in AFP cases and more investigation is needed for comparison with other virus strains with different clinical symptoms. *Echovirus* type 17 is not known to cause serious illness and has, therefore, attracted less attention than many other *Enteroviruses*.

It has been associated with sporadic cases of aseptic meningitis but not with severe neurological diseases. Moreover, it was isolated from the throat or rectal swabs of two patients with herpangina (30). A nation-wide outbreak has also been reported in the UK with a high proportion of patients with neurological symptoms including 2 with severe encephalitis and 1 death. This indicates that *Echovirus* type 17 is capable of spreading widely in the population and sometimes causing dangerous illnesses (31). It is noteworthy that isolation of a virus from feces only could not confirm its role in establishment of the disease because different types of *Enteroviruses* are often isolated from the feces of healthy individuals as well.

Although plenty of literature on serotypes of *Enteroviruses* is vailable, very limited information related to *Coxsackievirus* A8 characteristics and pathogenesis has been published. There was only one publication reporting its oncolytic effects on malignant human melanoma tumors and another article reporting its absence from fetuses or placentas of infected mice (32). A high proportion of children with *Enterovirus* infections, predominantly male, are reported (31). The same pattern was observed in this study with 71.4% (5 out of 7) male and 28.6% (2 out of 7) female. Moreover, according to the results of Laxmivandana et al. (33), higher infection rates in younger children, with the highest rates in those less than 2 years old, were demonstrated in this study.

The gold standard test for NPEV typing is the microneutralization assay (34). Nevertheless, this assay is labor intensive and time-consuming and the antisera for identification of all serotypes are not available in all laboratories. Moreover, the aggregation of viral particles, antigenic changes, and the multiplicity of viruses in one sample may lead to test failure. On the other hand, molecular typing methods work based on sequencing of the VP1 capsid protein gene and comparison of results with nucleotide or amino acid sequences of the prototype strains. This is a rapid, reliable and sensitive method to determine different strains of NPEVs (20). The present study showed the existence of *Adenovirus* and *Enterovirus* infections in AFPs with or without RP. One of the limitations of this study was the number of cases on which the study was performed. Although viral genome was detected in ≤ 25.44% of AFP cases, there was not a correlation between viral infection and paralysis. Comprehensive studies must be performed to obtain credible evidence to proceed with further theories about the role of viral infection in these patients. With the progress of the polio eradication program worldwide, a potent surveillance system should be dedicated to surveying the etiology and epidemic pattern of AFP. These kinds of studies provide a framework for further comprehensive investigations to provide the authorities with the information required to prevent exposure to or avoid risk factors involved in AFP incidence and prevalence. Despite lack of correlative evidence among AFP and the viral infection in the present survey, we suggest conducting this study with a bigger population size covering different countries to reach a better view on the correlation of different viral infections with AFPs specially RP positives with consideration to some immunology and genomic aspects of different populations in different countries.
